# Rare Association of Pneumomediastinum With Bilateral Pneumothorax

**DOI:** 10.7759/cureus.12091

**Published:** 2020-12-15

**Authors:** Noman Saleem, Rabia Saleem, Zara Saleem, Usman Ajmal

**Affiliations:** 1 Department of Forensic Medicine, Sahiwal Medical College, Sahiwal, PAK; 2 Neurosurgery, Punjab Institute of Neurosciences, Lahore, PAK; 3 Plastic Surgery, Lahore General Hospital, Lahore, PAK; 4 Pediatrics, Mayo Hospital, Lahore, PAK

**Keywords:** pneumomediastinum, pneumothorax, blunt thoracic trauma

## Abstract

Pneumomediastinum with bilateral pneumothorax is a clinical entity caused by infections, malignancy, or trauma, as in our case. Some patients present with pneumomediastinum secondary to trauma have esophageal, laryngeal, or tracheal injuries. A 16-year-old boy presented in the emergency department with complaints of shortness of breath and bruise on the chest after a history of the road traffic accident. Bilateral chest tube thoracotomy was done. Pneumomediastinum was suspected on X-ray chest and confirmed on computed tomography of the chest, which showed bilateral pneumothorax with pneumomediastinum. The patient was conservatively managed and discharged after 10 days.

## Introduction

Pneumothorax occurs when air leaks into the space between the lung and chest wall or within space between the visceral and parietal pleura, and pneumomediastinum is the presence of air in the mediastinum. Pneumomediastinum with pneumothorax is a rare condition, but when it occurs, it can be life-threatening, requiring immediate diagnosis and treatment. Pneumomediastinum is most commonly caused by blunt trauma and may occur in up to 10% of patients with severe blunt thoracic and cervical trauma [1]. Pneumomediastinum with bilateral pneumothorax is a rare clinical entity caused by infections, malignancy, or trauma, as in our case. There are only sporadic cases reported in the literature on this condition. In this case report, we describe a patient with pneumomediastinum associated with bilateral pneumothorax, who presented with subcutaneous emphysema and bruise on the chest along with difficulty in breathing without any identifiable cause.

## Case presentation

A 16-year-old boy of average built and height, a student of the ninth class, presented in the emergency department on the 3^rd^ of October, 2017, after he fell onto a parked rickshaw with shortness of breath. He had no past history of asthma or any other respiratory problems. On examination, there was tachycardia with a pulse rate of 110 beats per minute, hypotension with a blood pressure of 90/60, and a respiratory rate of 28 breaths/min. He had a bruise over his manubrium sterni and subcutaneous emphysema observed on his chest. On auscultation, breath sounds decreased on the right side of the chest. Heart sounds were regular. The rest of the examination was unremarkable.

**Figure 1:**
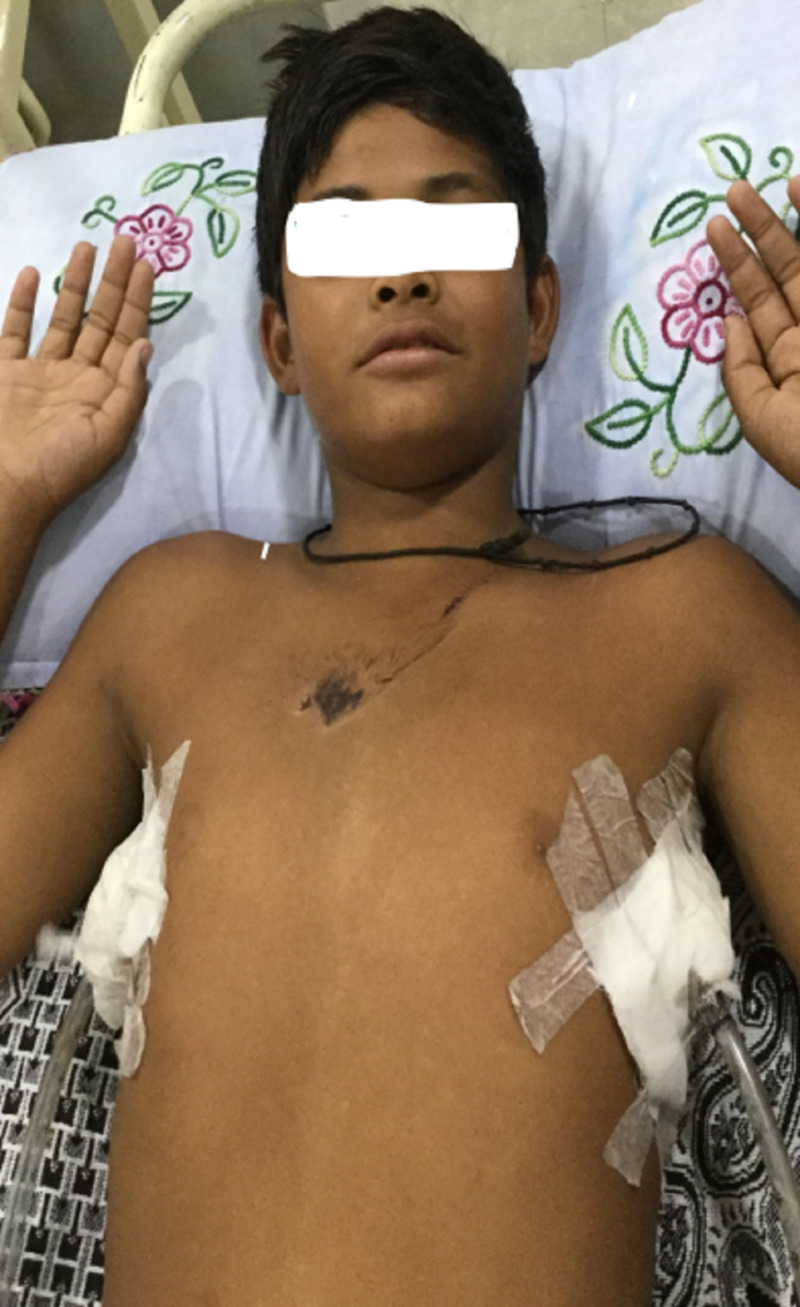
Chest tubes and bruise on the chest

Right-sided chest intubation was done for suspicion of right-sided tension pneumothorax. However, the patient didn’t improve, and his emphysema extended onto the neck and became very tense. Suspecting a tracheal injury, a left-sided thoracotomy was also done. A needle was also inserted in the neck and attached with an underwater seal to relieve the neck pressure. Both chest tubes were attached to negative suction pressure (Figure 1). The patient's vitals stabilized, and the needle in the neck was removed after one hour.

**Figure 2:**
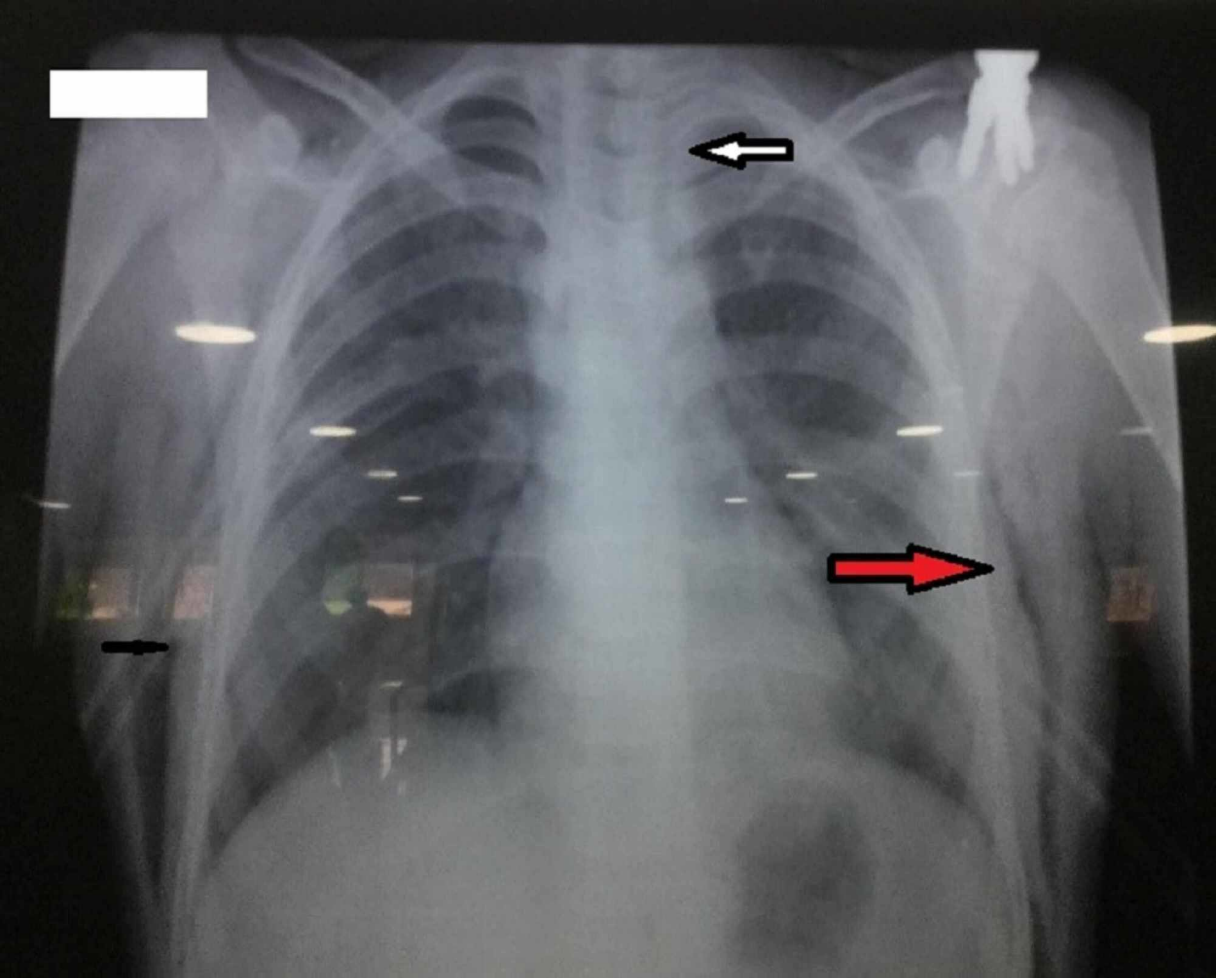
Chest X-ray posteroanterior view showing gas in the mediastinum (white arrow) and subcutaneous emphysema (red arrow)

The chest X-ray was done, which showed lucent streaks of gas that outlined mediastinal structures (Figure 2).

**Figure 3:**
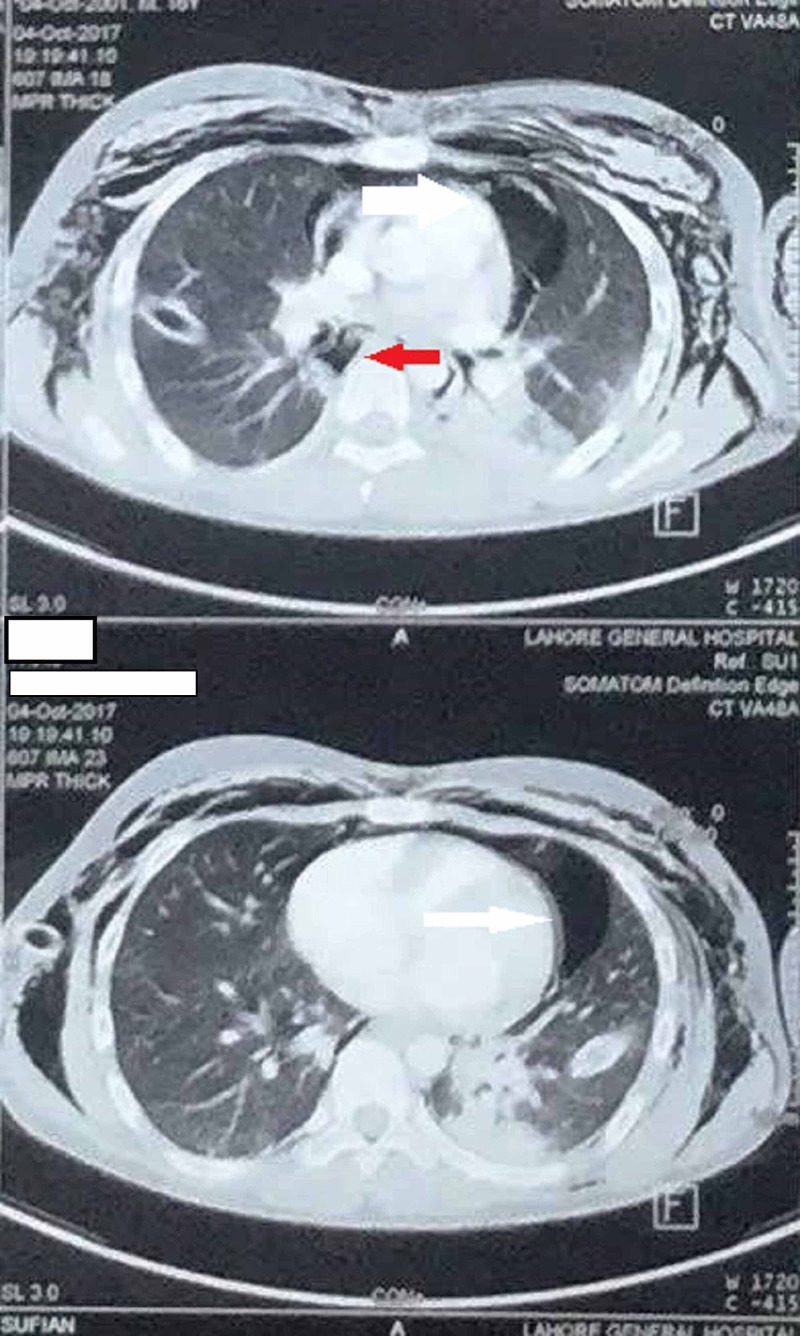
HRCT of the chest showing pneumothorax (white arrow) and pneumomediastinum (red arrow) HRCT - high-resolution CT

Computed tomography (CT) of the chest was ordered, which showed bilateral pneumothorax with pneumomediastinum (Figure 3).

The patient was managed conservatively with analgesics and a prophylactic course of antibiotics for 10 days. A bronchoscopy was planned, but the later decision was taken not to perform it as the patient settled with conservative management. Esophagoscopy was not performed as the patient had no gastrointestinal complaints. The patient was discharged after 10 days. He was instructed to avoid maneuvers that increase pulmonary pressure.

## Discussion

Subcutaneous emphysema is trapped air under the skin. Pneumothorax is defined as the air that accumulates into pleural space and causes the lung to collapse by expanding, and pneumomediastinum is air in the mediastinum. Pneumomediastinum with unilateral pneumothorax is a common condition. Pneumomediastinum can be categorized as atraumatic in around 20% or traumatic in around 80% of cases. Traumatic pneumomediastinum is caused by blunt trauma in around 86% or penetrating trauma in around 14% of cases or by iatrogenic injuries that are produced by mechanical ventilation or endoscopic procedures. About 10% of the patients with pneumomediastinum secondary to trauma have esophageal, laryngeal, or tracheal injuries [1, 2]. Looking at the non-iatrogenic tracheal injuries, the majority (64%) was caused by blunt chest trauma and the remaining by penetrating and bullet injuries [3].

Pneumothorax can be unilateral or bilateral and primary spontaneous (without underlying lung disease), secondary spontaneous (with underlying lung disease), or traumatic, as in our case. The most common cause of traumatic pneumomediastinum results from the so-called “Macklin effect” sudden increase in intrathoracic pressure, which results in increased intra-alveolar pressure leading to alveolar rupture, with air dissection along bronchovascular sheaths, and the spreading of this pulmonary interstitial emphysema into the mediastinum leading to alveolar rupture [2]. Esophageal injuries resulting from blunt trauma are rare events, occurring in <0.1% to 1.5% of patients [4]. Traumatic injury to the tracheobronchial tree is more common, occurring in up to 6% of patients [5].

The classical presentation is usually in patients with a history of varying degrees of shortness of breath, chest pain, difficulty swallowing, and subcutaneous emphysema (crepitation on touch), but our patient came to us with dyspnea. Physical examination of about 30% of patients can be normal in pneumomediastinum [3]. Usually, there may be tachycardia, tachypnea, hyper resonance to percussion, diminished breath sounds, and asymmetrical chest wall expansion may be present on physical examination. Distended neck veins, hypotension or cyanosis, and swelling on the chest radiate to the neck and face mostly due to underlying tension pneumothorax [4]. As in the presented case, the patient is hypotensive, tachycardiac, decreased breath sounds on the right side of the chest initially, and swelling on the chest and neck. However, diagnosis depends on the history and physical examination of the patient but can be confirmed by X-ray, CT of the chest, bronchoscopy for tracheal injuries, esophagoscopy for esophageal lacerations [6]. To make a correct diagnosis, frontal and lateral views of chest radiographs are needed. Accurate interpretation of the chest radiograph is essential in the early diagnosis of the occult upper-airway injury. Radiographic signs of pneumomediastinum on the X-ray of the chest include lucent streaks of gas that outlines mediastinal structures. The reported case was confirmed by X-ray and high-resolution tomography (HRCT) of the chest. Although bronchoscopy and esophagoscopy should be done to rule out any aerodigestive tract injuries, we did not perform it after consultation with a pulmonologist and gastroenterologist due to improvement in patient condition [7]. This is the major limitation in this case report. Whenever bilateral pneumothorax is suspected with pneumomediastinum, initial multidisciplinary evaluation is important for management. Bilateral pneumothorax can be treated according to age, clinical status, and underlying causes. It is usually benign in young patients and can be life-threatening in advanced ages and in patients with limited pulmonary reserve, requiring urgent management. Missed diagnosis and delayed treatment can lead to tension pneumothorax and the patient’s death [8, 9]. Aerodigestive tract injuries can be managed conservatively or by surgery, depending on patients' clinical status [3, 7]. Surgery is recommended for clinically unstable patients, as most patients with major aerodigestive tract injuries undergo primary repair. Pneumonia and mediastinitis prophylaxis should be given to all patients. Our patient became stable with bilateral chest tube thoracotomy and antibiotic cover, and the vitally stable patient was discharged after 10 days.

## Conclusions

Pneumomediastinum with bilateral pneumothorax is a rare clinical condition caused by trauma and needs a multidisciplinary approach for early management and stabilization of the patient. Early diagnosis is vital for saving the life of the patient. It can be diagnosed only with meticulous clinical examination and radiological studies. Although management is mostly conservative in a critical care setting, surgical interventions may sometimes be required along with careful follow-up.
